# Timing, rates, and causes of death in a large South African tuberculosis programme

**DOI:** 10.1186/s12879-014-0679-9

**Published:** 2014-12-21

**Authors:** Nigel Field, Megan SC Lim, Jill Murray, Robert J Dowdeswell, Judith R Glynn, Pam Sonnenberg

**Affiliations:** Research Department of Infection and Population Health, University College London, Mortimer Market Centre (off Capper St), London, WC1E6JB UK; Centre for Population Health, Burnet Institute, Melbourne, Australia; National Institute for Occupational Health, National Health Laboratory Service and School of Public Health, University of the Witwatersrand, Johannesburg, South Africa; Rustenburg Platinum Mines Limited, Rustenburg, South Africa; Department of Infectious Disease Epidemiology, London School of Hygiene and Tropical Medicine, London, UK

**Keywords:** Tuberculosis mortality, Tuberculosis treatment, HIV, Antiretroviral therapy (ART), Autopsy

## Abstract

**Background:**

Tuberculosis (TB) mortality remains high across sub-Saharan Africa despite integration of TB and HIV/ART programmes. To inform programme design and service delivery, we estimated mortality by time from starting TB treatment.

**Methods:**

Routinely collected data on TB treatment, vital status, and the timing and causes of death, were linked to cardio-respiratory autopsy data, from 1995–2008, from a cohort of male platinum miners in South Africa. Records were expanded into person-months at risk (pm).

**Results:**

4162 TB episodes were registered; 3170 men were treated for the first time and 833 men underwent retreatment. Overall, 509 men died, with a case fatality of 12.2% and mortality rate of 2.0/100 pm. Mortality was highest in the first month after starting TB treatment for first (2.3/100 pm) and retreatment episodes (4.8/100 pm). When stratified by HIV status, case fatality was higher in HIV positive men not on ART (first episode 14.0%; retreatment episode 26.2%) and those on ART (12.0%; 22.0%) than men of negative or unknown HIV status (2.6%; 3.6%). Mortality was also highest in the first month for each of these groups. Mortality risk factors included older age, previous TB, HIV, pulmonary TB, and diagnostic uncertainty. The proportion of deaths attributable to TB was consistently overestimated in clinical records versus cardio-respiratory autopsy.

**Conclusions:**

Programme mortality was highest in those with HIV and during the first month of TB treatment in all groups, and many deaths were not caused by TB. Resource allocation should prioritise TB prevention and accurate earlier diagnosis, recognise the role of HIV, and ensure effective clinical care in the early stages of TB treatment.

**Electronic supplementary material:**

The online version of this article (doi:10.1186/s12879-014-0679-9) contains supplementary material, which is available to authorized users.

## Background

In South Africa, tuberculosis (TB) is the most commonly reported cause of death in adults, and most TB-deaths are associated with HIV-infection [1],[2]. Despite dedicated TB programmes and their integration with HIV programmes as antiretroviral treatment (ART) is increasingly available across sub-Saharan Africa (SSA), mortality within TB programmes remains high in many settings [2]-[6]. Detailed understanding of the timing and causes of mortality provides a focus for the planning of services and interventions.

TB mortality tends to be highest in the first two months on TB treatment [7]-[11], and early mortality is increased in specific patient-groups, including older people and those with previous TB episodes, smear-negative TB and extra-pulmonary TB [9]. HIV co-infection increases the TB mortality rate, and HIV positive patients continue to die throughout TB treatment, whereas mortality in HIV negative patients declines rapidly after the first month of treatment [5],[7],[12]-[15]. While ART improves outcomes for HIV-positive TB patients overall, the mortality rate during the first one to two months of TB treatment for HIV-infected patient groups seems not to be reduced by ART [11],[16]. This could reflect selection of those most immunocompromised for ART or the impact of immune reconstitution inflammatory syndrome (IRIS) on survival [11],[16].

Only two studies describing the cause of death in TB patients by time on treatment have verified cause of death using autopsy, both prior to the introduction of ART [17],[18]. These suggest that most deaths in the early stages of treatment are attributable to TB, whereas those in the later stages of treatment usually have other causes [4],[5],[14],[17]-[19]. The cause of death also differs by HIV status in TB programmes, with most deaths in HIV negative patients attributable to TB, whereas HIV positive patients die from a variety of infectious causes [5],[18].

However, the available data are limited by relatively small patient numbers, and no single study has investigated the timing and causes of deaths among first and re-treatment episodes while stratifying by HIV status and whether patients have received ART. Furthermore, although we know that clinical misclassification of deaths in TB programmes is common [16],[20],[21], there is a paucity of studies using autopsy to confirm the cause of death [22],[23].

We have previously reported data from a South African platinum mining company, following a well-defined population with a high burden of TB (1,866 per 100,000 person years between 1995–2008) and HIV to show the impact of HIV, ART and TB on population mortality [3], and have described measurement errors in estimating TB-specific mortality [21]. Here, we focus on mortality at a programme level by time from starting TB treatment in a large cohort, including patients on ART and using multiple data sources to determine the timing and cause of death with a high degree of precision.

## Methods

The study setting was the TB treatment programme of a platinum mine in North West Province, South Africa. Medical care is provided free of charge by the company and includes primary care, hospital facilities, and an HIV programme providing ART since April 2003 [24]. Miners diagnosed with TB received treatment in accordance with South African National TB guidelines at the time, which recommended regimen 1 for new adult TB patients (comprising two months of rifampicin (R), isoniazid (H), pyrazinamide (Z), ethambutol (E), followed by four months of RH) and regimen 2 for retreatment cases (comprising two months of RHZE and streptomycin, one month of RHZE, and five months of RHE) [25]. HIV-positive employees are registered on the ART programme, which monitors CD4 counts, clinical status and, where appropriate, the mining company provides free ART. Patients were eligible for ART with a CD4 count of <250 cells/μl, WHO stage 4, or WHO stage 3 with a CD4 count of <350 cells/μl. All semiskilled and unskilled male miners registered on the TB programme were selected for this study because it was possible to link these individuals across TB programme, HIV/ART programme, death register, autopsy register, and company databases using unique employee identification codes.

Routine TB-programme data were available for TB episodes with treatment initiated between 1 January 1995 and 31 December 2008. We included all diagnosed TB episodes, regardless of site of TB (pulmonary, extra-pulmonary, or unknown) and of bacteriological confirmation, to assess overall mortality at a programme level. Sputum smear and culture data were only available after 2000, and other clinical data, including antibiotic resistance, were unavailable. We classified TB cases as “confirmed” if at least one sputum culture was positive for *Mycobacterium tuberculosis*, “probable” if at least one sputum smear was positive for acid-fast bacilli, and “possible” if there was at least one negative sputum culture and/or sputum smear. The remaining cases lacked microbiological data and were treated as a separate “no data” category. TB episodes were classified as first episode unless previous episodes were documented during the study period or recorded in the programme database. Men were classified as HIV positive if they had a recorded HIV positive test; if they were identified as HIV positive through the TB or ART programme databases; if their medical record or death certificate stated HIV, AIDS or a euphemistic term such as ‘immune compromised’; or if their cause of death was obviously an AIDS-related condition (*Pneumocystis jirovecii* pneumonia (PCP), aspergillus, nocardia pneumonia, cryptococcal disease, oesophageal candidiasis, lymphadenopathy, lymphocytic interstitial pneumonitis, cytomegalovirus pneumonia, toxoplasmosis, or Kaposi’s sarcoma) [3]. HIV testing dates and HIV status at death were not routinely recorded, and HIV negative results only rarely recorded, so men not known to be positive were classified as ‘HIV negative or unknown’. 97 men (6 originally classed as HIV negative and 91 as HIV status unknown) were reclassified as HIV positive because their cause of death was clearly AIDS-related. Men were classified as being on ART if this was recorded by TB or ART programme databases. CD4 counts, HIV viral load and disease stage were not available for most men in the cohort.

The timing and cause of death were the primary outcomes. Men entered the cohort on the day of initiating TB treatment and remained in the cohort for six months after this date for first TB episodes and for eight months for retreatment episodes, and were otherwise censored only when they died within these time periods. Our rationale here was to understand overall programme mortality during the standard periods of treatment, when most deaths occur. We know that data on hospital transfer and treatment default were incomplete and susceptible to misclassification, but timing of death is extremely reliable in this study due to the short follow-up time, and availability of multiple data sources used to ascertain date of death. These included the hospital death register, personnel records, ART and TB programme records, and the provident fund records (which record deaths for compensation purposes for twelve months after men leave the mine). Autopsies of cardio-respiratory organs were conducted on miners for compensation purposes, with the consent of next of kin, and regardless of the clinical cause of death. The lungs are removed at the place of death, placed in formalin and sent to the National Institute for Occupational Health (NIOH) in Johannesburg where they are examined macroscopically and histologically according to a standard protocol by anatomical pathologists with experience in lung pathology. At least six tissue sections from the upper, middle and lower zones of both the right and left lungs, as well as sections of one main bronchus and two hilar lymph nodes are routinely processed for histology. Where there is any clinical or pathological evidence of infection, tissue sections are stained with alcian-blue (for cryptococcus), Ziehl-Neelsen (for mycobacteria) and Grocott (for Pneumocystis and other fungi), in addition to haematoxylin and eosin. Infections are diagnosed according to standard histological criteria [26]. Briefly, bacterial pneumonia is defined as the presence of consolidation with polymorphonuclear leukocytes in alveolar spaces, in the absence of positive stains for mycobacteria, fungi, pneumocystis, cryptococci, nocardia or other organisms. Tuberculosis is diagnosed 1) in the presence of granulomatous inflammation with positive Ziehl-Neelsen stains or 2) in the presence of granulomatous inflammation, other causes having been excluded by special stains and 3) cognisance is taken of atypical reactions to mycobacteria which may occur in immune-compromised patients such as poorly formed granulomas, sarcoidal-like granulomas, bland necrosis and neutrophil rich exudates. We compared the clinical cause of death to cardio-respiratory autopsy data obtained from the NIOH autopsy database.

Case fatality was calculated for each time period, and records were expanded into person months at risk (pm), by time from starting TB treatment, to calculate mortality rates per 100 pm with 95% CI. Incidence rate ratios (IRR) were used to quantify differences in mortality rates with 95% CI, using Poisson regression. Risk factors for dying were identified using Poisson regression, separately for the first month of treatment and later months in univariable and multivariable analyses, with age and HIV/ART status as time-varying covariates. Chi-squared tests were used to compare characteristics of those autopsied and those not. Kaplan-Meier survival analysis was used to generate survival curves and a log rank test was used to compare survival between groups. All analyses were conducted using Stata 11 (Stata Corp, College Station, TX, USA).

Ethical approval was obtained from the Human Research Ethics Committee (Medical) of the University of the Witwatersrand, South Africa (M041028), and the UCL Research Ethics Committee, United Kingdom (1641/001). The Committees specifically addressed issues of consent and confidentiality. Given the retrospective nature of the study; high mortality; use of routine databases linked by a unique number; and removal of personal identifiers prior to analysis, specific written consent for inclusion in this research was not required. Autopsies were conducted with written consent from the next of kin, in accordance with the South African Occupational Diseases in Mines and Works Act. The project is covered by the UCL Data Protection Registration (Reference No Z6364106/2008/8/18, Section 19, Research: Health Research).

## Results

During 1995 to 2008, there were 4162 treated TB episodes recorded by the mine hospital TB programme. Table 1 shows that the majority were in employment for at least ten years, aged 35–54 years at first episode, and diagnosed with pulmonary TB. 3170 men had a first episode of TB, of whom 2046 (65%) were HIV positive. There were 992 retreatment episodes in 833 men, of which 775 episodes (78%) were in HIV positive men.

**Table 1:** **Patient characteristics for 4,162 TB episodes occurring between 1995 and 2008**

		First episode n (%)	Retreatment episode n (%)
TOTAL		3170	992
Age at diagnosis	<35 years	438 (14)	86 (9)
	35-44 years	1208 (38)	364 (37)
	45-54 years	1261 (40)	470 (47)
	>54 years	263 (8)	72 (7)
Treatment sequence	First episode	3170 (100)	
	Second episode		833 (84)
	Third episode		140 (14)
	Fourth or fifth episode		19 (2)
Time in employment at diagnosis	<1 year	237 (7)	27 (3)
	1- < 5 years	526 (17)	116 (12)
	5- < 10 years	461 (15)	149 (15)
	10+ years	1946 (61)	700 (71)
Year diagnosed	1995-1998	509 (16)	234 (24)
	1999-2002	848 (27)	330 (33)
	2003-2005	908 (29)	290 (29)
	2006-2008	905 (29)	138 (14)
HIV/ART status	Negative/unknown	1124 (36)	217 (22)
	Positive: ART	375 (12)	277 (28)
	Positive: no ART	1671 (53)	498 (50)
TB site	Pulmonary	1706 (54)	687 (69)
	Extra-pulmonary^1^	1276 (40)	259 (26)
	No data	188 (6)	46 (5)
Certainty of diagnosis^2^	Confirmed	740 (23)	269 (27)
	Probable	760 (24)	314 (32)
	Possible	856 (27)	264 (27)
	No data	814 (26)	145 (15)
Outcome	Cured/Completed	2362 (75)	589 (59)
	Failed	54 (2)	25 (3)
	Did not complete	170 (5)	41 (4)
	Transferred	276 (9)	136 (14)
	Died	308 (10)	201 (20)
Autopsy data available	Alive	2862 (90)	791 (80)
	Died – Cardiorespiratory autopsy	180 (6)	113 (11)
	Died – No autopsy	128 (4)	88 (9)

^1^Extra-pulmonary cases were in the following sites: 59 lymph node, 489 pleural, 21 meningitis, 65 bone/joint, 124 other organs, 77 miliary.

^2^TB cases were classified as “confirmed” if at least one sputum culture was positive for *Mycobacterium tuberculosis*, “probable” if at least one sputum smear was positive for acid-fast bacilli, and “possible” if there was at least one negative sputum culture and/or sputum smear. The remaining cases lacked microbiological data and were treated as a separate “no data” category.

Overall, 509 men died, with a case fatality of 12.2% and mortality rate of 2.0/100 pm (Table 2). Mortality was highest in the first month of treatment for both first (2.3/100 pm) and retreatment TB episodes (4.8/100 pm). First episode mortality rates were lower than retreatment mortality rates at each comparable month. By the end of the sixth month after starting TB treatment, the cumulative case fatality was 9.7% for first episodes and 17.6% for retreatment episodes. The mortality rate remained stable from the second month for first TB episodes, whereas the mortality rate declined throughout retreatment.

**Table 2:** **Case fatality, mortality rate and risk of death by month since starting TB treatment, by first and retreatment TB episodes**

	Time on treatment	n	Number of deaths	Cumulative case fatality (%)	Person months	Rate per 100 pm (95% CI)	IRR (95% CI)	*p* -value
First episode	1st month	3170	71	2.2	3132	2.3 (1.8-2.9)	1.0	
	2nd month	3099	50	3.8	3072	1.6 (1.2-2.1)	0.72 (0.50-1.0)	0.07
	3rd month	3049	47	5.3	3023	1.6 (1.2-2.1)	0.69 (0.47-0.99)	0.05
	4th month	3002	46	6.8	2979	1.5 (1.2-2.1)	0.68 (0.47-0.99)	0.04
	5th month	2956	44	8.1	2936	1.5 (1.1-2.0)	0.66 (0.45-0.96)	0.03
	6th month	2912	50	9.7	2889	1.7 (1.3-2.3)	0.76 (0.53-1.10)	0.14
	Overall	3170	308	9.7	18030	1.7 (1.5-1.9)		
Retreatment episode	1st month	992	46	4.6	967	4.8 (3.6-6.4)	1.0	
	2nd month	946	28	7.5	933	3.0 (2.1-4.3)	0.63 (0.39-1.01)	0.05
	3rd month	918	34	10.9	898	3.8 (2.7-5.3)	0.80 (0.51-1.24)	0.31
	4th month	884	32	14.1	865	3.7 (2.6-5.2)	0.78 (0.50-1.22)	0.28
	5th month	852	19	16.0	844	2.3 (1.4-3.5)	0.47 (0.28-0.81)	0.01
	6th month	833	16	17.6	827	1.9 (1.2-3.2)	0.41 (0.23-0.72)	<0.01
	7th month	817	17	19.4	808	2.1 (1.3-3.4)	0.44 (0.25-0.77)	<0.01
	8th month	800	9	20.3	793	1.1 (0.6-2.2)	0.24 (0.12-0.49)	<0.01
	Overall	992	201	20.3	6935	2.9 (2.5-3.3)		
Overall		4162	509	12.2	24965	2.0 (1.9-2.2)		

Among first TB episodes (Table 3), case fatality was lower in men with negative or unknown HIV status (2.6%) than HIV positive men on ART (12.0%) and HIV positive men not on ART (14.0%). In each group, mortality was highest in the first month of treatment, and decreased later, although not to the same extent in all groups. Within the group on ART, overall mortality was 2.1/100 pm, and was not significantly different to the group not on ART (2.5/100 pm). There was also no significant difference in mortality between the 208 men who started ART within one month of starting TB treatment (2.0/100 pm (1.3-2.9)) and the 167 men who were treated with ART for longer than one month when starting TB treatment (2.4/100 pm (1.6-3.6)).

**Table 3:** **Case fatality, mortality rate and risk of death by month since starting TB treatment for first TB episodes, by HIV status**

HIV/ART status	Time on treatment ^1^	n	Number of deaths	Cumulative case fatality (%)	Person months	Rate per 100 pm (95% CI)	IRR (95% CI)	p-value for trend
								<0.01
HIV negative or unknown	1st month	1124	10	0.9	1117	0.9 (0.5-1.7)	1.0	
	2nd month	1114	8	1.6	1109	0.7 (0.4-1.4)	0.81 (0.32-2.04)	
	3rd-6th month	1106	11	2.6	4401	0.2 (0.1-0.5)	0.28 (0.12-0.66)	
	Overall	1124	29	2.6	6627	0.4 (0.3-0.7)		
								0.98
HIV positive on ART	1st month	375	15	4.0	367	4.1 (2.5-6.8)	1.0	
	2nd month	360	2	4.5	358	0.6 (0.1-2.2)	0.14 (0.03-0.60)	
	3rd-6th month	358	28	12.0	1372	2.0 (1.4-3.0)	0.50 (0.27-0.94)	
	Overall	375	45	12.0	2097	2.1 (1.6-2.9)		
								0.22
HIV positive not on ART	1st month	1671	46	2.8	1647	2.8 (2.1-3.7)	1.0	
	2nd month	1625	40	5.1	1604	2.5 (1.8-3.4)	0.89 (0.58-1.36)	
	3rd-6th month	1585	148	14.0	6054	2.4 (2.1-2.9)	0.88 (0.63-1.22)	
	Overall	1671	234	14.0	9306	2.5 (2.2-2.9)		

^1^Months 3–6 were grouped because mortality rates were observed to be stable in new TB episodes during this time period and the numbers of deaths were small.

**Figure 1:**
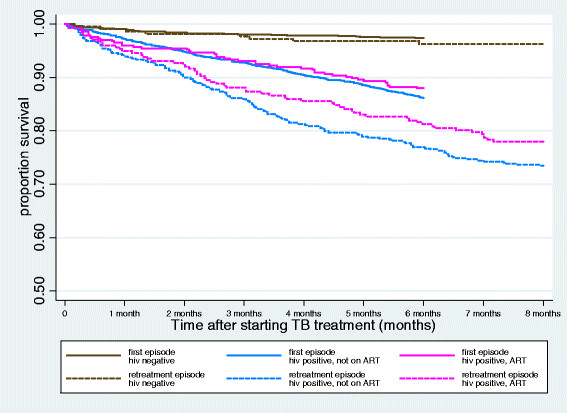
**Kaplan-Meier curves showing survival time for first and retreatment TB episodes, by HIV status.**

Mortality by month in HIV positive men, whether or not on ART, was considerably higher during retreatment than during first TB episodes, whereas monthly mortality in men who were HIV negative or of unknown status was similar during first and retreatment episodes (Tables 3 and 4). Nevertheless, the overall trends in monthly case fatality and mortality rates by HIV status among men undergoing TB retreatment were similar to the trends we observed for first episodes, with the highest mortality in the first month of treatment for all groups. Kaplan-Meier curves show differences in survival by first and retreatment episodes and according to HIV status (log rank test p < 0.01) (Figure 1).

**Table 4:** **Case fatality, mortality rate and risk of death by month since starting TB treatment for retreatment TB episodes, by HIV status**

HIV/ART status	Time on treatment ^1^	n	Number of deaths	Cumulative case fatality (%)	Person months	Rate per 100 pm (95% CI)	IRR (95% CI)	p-value for trend
								0.06
HIV negative or unknown	1st month	217	2	0.9	216	0.9 (0.2-3.7)	1.0	
	2nd month	215	2	1.8	213	0.9 (0.2-3.7)	1.01 (0.14-7.18)	
	3rd-6th month	213	4	3.7	844	0.5 (0.2-1.3)	0.51 (0.09-2.79)	
	7th-8th month	209	0	3.7	418	0.0	--	
	Overall	217	8	3.7	1691	0.5 (0.2-0.9)		
								<0.01
HIV positive on ART	1st month	277	14	5.1	270	5.2 (3.1-8.8)	1.0	
	2nd month	263	7	7.6	259	2.7 (1.3-5.7)	0.52 (0.21-1.29)	
	3rd-6th month	256	31	18.8	951	3.3 (2.3-4.6)	0.63 (0.33-1.18)	
	7th-8th month	225	9	22.0	439	2.1 (1.1-3.9)	0.39 (0.17-0.91)	
	Overall	277	61	22.0	1919	3.2 (2.5-4.1)		
								0.03
HIV positive not on ART	1st month	498	30	6.0	482	6.2 (4.4-8.9)	1.0	
	2nd month	468	19	9.8	461	4.1 (2.6-6.5)	0.66 (0.37-1.18)	
	3rd-6th month	449	66	23.1	1639	4.0 (3.2-5.1)	0.65 (0.42-1.00)	
	7th-8th month	383	17	26.5	744	2.3 (1.4-3.7)	0.37 (0.20-0.66)	
	Overall	498	132	26.5	3325	4.0 (3.3-4.7)		

^1^Months 3–6 and 7–8 were grouped because mortality rates were observed to be stable during these time periods and the numbers of deaths were small.

**Figure 2:**
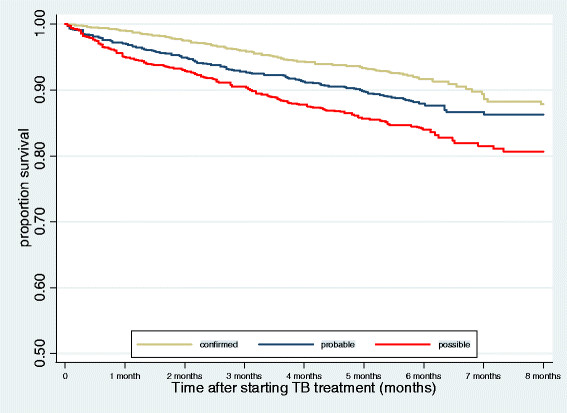
**Kaplan-Meier curves showing survival time for TB episodes with microbiological data, by certainty of diagnosis.**

We analysed risk factors associated with death for first and later months of treatment (Table 5). For both time periods, in univariable analysis, mortality was associated with older age, previous TB, HIV infection, pulmonary TB, and certainty of diagnosis. Mortality was higher in more recent calendar periods in the first month of treatment, but not in later months. Compared to the unadjusted models, the findings were similar in multivariable analyses. The exception was that we observed a large increase in the risk of death associated with having no smear or culture data in the adjusted model, and we found this was due to the confounding effect of year of diagnosis. A strong effect of calendar period remained in the multivariable model.^3^ For HIV positive men on ART compared to those not on ART, the adjusted IRR was lower in the first month (IRR 0.86 (0.55-1.36)) and in subsequent months (IRR 0.77, 0.58-1.01), although not significantly so. Kaplan-Meier curves show survival by certainty of TB diagnosis, excluding episodes without smear or culture data (Figure 2). In the first and later months of treatment, those with confirmed TB (positive culture) were less likely to die than those with probable TB (positive smear but negative or absent culture), and those with possible TB (> = one negative smear or culture) (log rank test p < 0.01).

**Table 5:** **Risk factors for death stratified by time since starting TB treatment (includes first and retreatment episodes up to six months)**

			First Month					Later months (2–6)				
	Category	n	Number of deaths	Unadjusted IRR (95% CI)	*p* -value	Adjusted ^#^	*p* -value	Number of deaths	Unadjusted IRR (95% CI)	*p* -value	Adjusted ^#^	*p* -value
						IRR (95% CI)					IRR (95% CI)	
Age at diagnosis*	<35 years	524	8	1.0	0.06	1.0	0.17	27	1.0	<0.01	1.0	<0.01
	35-44 years	1572	38	1.59 (0.74-3.41)		1.57 (0.73-3.37)		144	1.70 (1.12-2.59)		1.53 (1.01-2.33)	
	45-54 years	1731	58	2.19 (1.05-4.59)		1.92 (0.91-4.05)		182	1.98 (1.31-2.98)		1.77 (1.17-2.68)	
	>54 years	335	13	2.53 (1.05-6.10)		2.43 (1.00-5.88)		39	2.65 (1.65-4.27)		2.68 (1.66-4.33)	
TB episode	First	3170	71	1.0	<0.01	1.0	<0.01	237	1.0	<0.01	1.0	<0.01
	> = 1 previous	992	46	2.10 (1.45-3.04)		2.20 (1.49-3.26)		155	1.63 (1.33-2.00)		1.46 (1.17-1.82)	
HIV/ART status*	Negative/unknown	1341	12	1.0	<0.01	1.0	<0.01	25	1.0	<0.01	1.0	<0.01
	Positive: ART	652	29	5.06 (2.59-9.91)		3.17 (1.56-6.44)		77	6.37 (4.05-10.00)		6.02 (3.75-9.67)	
	Positive: no ART	2169	76	3.97 (2.16-7.29)		3.59 (1.94-6.66)		290	7.71 (5.13-11.61)		7.80 (5.15-11.80)	
TB site	Pulmonary	2393	77	1.0	0.02	1.0	<0.01	260	1.0	<0.01	1.0	<0.01
	Extra-pulmonary	1535	30	0.60 (0.40-0.92)		0.54 (0.33-0.91)		115	0.69 (0.56-0.86)		0.57 (0.42-0.77)	
	No data	234	10	1.35 (0.70-2.60)		1.41 (0.69-2.88)		17	0.68 (0.42-1.12)		0.76 (0.46-1.27)	
Year first diagnosed	1995-1998	742	9	1.0	<0.01	1.0	<0.01	58	1.0	<0.01	1.0	0.81
	1999-2002	1177	24	1.69 (0.78-3.63)		1.81 (0.79-4.17)		129	1.47 (1.07-2.01)		1.18 (0.82-1.68)	
	2003-2005	1198	46	3.21 (1.57-6.55)		3.75 (1.63-8.61)		118	1.36 (0.99-1.87)		1.14 (0.77-1.69)	
	2006-2008	1043	38	3.05 (1.47-6.30)		3.97 (1.73-9.11)		87	1.18 (0.85-1.65)		1.14 (0.76-1.71)	
Certainty of diagnosis	Confirmed	1009	10	1.0	<0.01	1.0	<0.01	84	1.0	0.07	1.0	<0.01
	Probable	1074	32	3.05 (1.50-6.20)		3.38 (1.65-6.93)		102	1.17 (0.88-1.56)		1.17 (0.87-1.58)	
	Possible	1120	56	5.15 (2.63-10.09)		6.28 (3.18-12.43)		134	1.56 (1.19-2.05)		1.73 (1.30-2.30)	
	No data	959	19	2.01 (0.94-4.33)		9.06 (3.82-21.51)		72	0.95 (0.70-1.31)		2.51 (1.64-3.85)	

*time-varying covariates.

^#^Models were adjusted for all factors in the table.

Cardio-respiratory autopsy data were available for 121/228 (53%) of deaths occurring in men with confirmed or probable TB and 106/190 (56%) of deaths with possible TB (Tables 6 and 7)). The proportion of deaths for which autopsy was performed decreased from 94% in 1997 to 39% in 2008 (*p* < 0.01), but men undergoing autopsy were similar to those not autopsied according to HIV prevalence (92% in autopsied and 94% in not autopsied men (*p* = 0.6)) and age (mean 45.5 years in autopsied and 44.9 years in men not autopsied men (*p* = 0.2)). Clinical cause of death overestimated the proportion of deaths attributable to TB in all groups, particularly in the latter stages of treatment in HIV positive men. For example, among HIV positive men not on ART in the later months of treatment, TB was recorded as the clinical cause of death in 62% of deaths, whereas only 26% of deaths could be attributed to TB at autopsy (Table 6). Other infectious causes, often not identified in life, accounted for many deaths at later time points and in HIV positive men. Pneumonia, PCP, and other infections were the leading causes among non-TB natural deaths. At autopsy, overall, only 35% of deaths could be attributed to TB in men with confirmed, probable or possible TB dying within six months of starting treatment (Tables 6 and 7).

**Table 6:** **Clinical and autopsy cause of death by month since starting TB treatment for 228 deaths among men with confirmed and probable TB** ^**1**^ **, by HIV status, n (%)**

	HIV negative or unknown			HIV positive on ART			HIV positive not on ART			TOTAL
	1st month	2nd-6th month	TOTAL	1st month	2nd-6th month	TOTAL	1st month	2nd-6th month	TOTAL	
Clinical Cause of Death										
TB	1 (50)	6 (75)	7 (70)	8 (100)	25 (66)	33 (72)	21 (66)	87 (62)	108 (63)	148 (65)
Non-TB natural deaths	1 (50)	2 (25)	3 (30)	0	13 (34)	13 (28)	11 (34)	52 (37)	63 (37)	79 (35)
Unnatural deaths	0	0	0	0	0	0	0	1 (1)	1 (1)	1 (0)
Autopsy Cause of Death										
TB	1 (50)	2 (50)	3 (50)	3 (60)	6 (33)	9 (39)	11 (58)	19 (26)	30 (33)	42 (35)
Non-TB natural deaths	1 (50)	2 (50)	3 (50)	2 (40)	12 (67)	14 (61)	8 (42)	54 (73)	61 (66)	78 (64)
*Pneumonia*			*2*	*2*	*5*	*7*	*1*	*19*	*20*	*29*
*Pneumocystis pneumonia*			*0*		*2*	*2*	*2*	*13*	*15*	*17*
*Cryptococcus*			*0*			*0*	*1*	*6*	*7*	*7*
*Other infection* ^*2*^			*0*		*4*	*4*	*1*	*8*	*9*	*13*
*Kaposi’s sarcoma*			*0*			*0*		*1*	*1*	*1*
*Lung cancer*			*0*		*1*	*1*	*1*	*1*	*2*	*3*
*Other cancer*	*1*	*2*	*1*			*0*	*1*	*1*	*2*	*3*
*Other medical* ^*3*^			*0*			*0*	*1*	*4*	*5*	*5*
Unnatural deaths			0			0		1 (1)	1 (1)	1 (1)
*No autopsy done*	*0*	*4*	*4*	*3*	*20*	*23*	*13*	*67*	*80*	*107*

^1^228 men included 54 treated for the first time and 40 retreated with confirmed TB, and 75 treated for the first time and 59 retreated with probable TB (see Table 1 footnote 1 for definitions used).

^2^Includes 12 men with HIV infection not otherwise specified and one with HIV infection and meningitis as the cause of death.

^3^Includes three men with stroke, one with diabetes, and one with silicosis as the cause of death.

Italics give numbers of deaths without percentages.

**Table 7:** **Clinical and autopsy cause of death by month since starting TB treatment for 190 deaths among men with possible TB** ^**1**^ **, by HIV status, n (%)**

	HIV negative or unknown			HIV positive on ART			HIV positive not on ART			TOTAL
	1st month	2nd-6th month	TOTAL	1st month	2nd-6th month	TOTAL	1st month	2nd-6th month	TOTAL	
Clinical Cause of Death										
TB	3 (50)	1 (33)	4 (44)	10 (56)	23 (61)	33 (59)	18 (56)	58 (62)	76 (61)	113 (59)
Non-TB natural deaths	2 (33)	2 (67)	4 (44)	8 (44)	14 (37)	22 (39)	14 (44)	34 (37)	48 (38)	74 (39)
Unnatural deaths	1 (17)	0	1 (11)	0	1 (3)	1 (2)	0	1 (1)	1 (1)	3 (2)
Autopsy Cause of Death										
TB	1 (33)	0	1 (25)	5 (36)	8 (44)	13 (41)	6 (35)	17 (32)	23 (33)	37 (35)
Non-TB natural deaths	1 (33)	1 (100)	1 (50)	9 (64)	10 (56)	19 (59)	11 (65)	36 (68)	47 (67)	68 (64)
Unnatural deaths	1 (33)	0	1 (25)	0	0	0	0	0	0	1 (1)
*No autopsy done*	*3*	*2*	*5*	*4*	*20*	*24*	*15*	*40*	*55*	*84*

^1^190 men included 118 treated for the first time and 72 retreated with possible TB (see Table 1 footnote 1 for definitions used).

Italics give numbers of deaths without percentages.

## Discussion

This study quantifies case fatality and mortality rates by time from starting TB treatment within a large TB programme in South Africa over fourteen years. The strengths of this study lie in the large numbers, precision with which the occurrence and timing of deaths were ascertained, and availability of cardiorespiratory autopsy data. For the first time in the ART era, we quantify the high risk of death during the first month of TB treatment at a programme level, and emphasise the risks associated with retreatment and HIV status. Retreated men were more than twice as likely to die as men undergoing a first TB episode, and HIV positive men were over three times more likely to die than men of negative or unknown HIV status. These data may inform projected estimates of mortality among co-infected patients and corresponding lives saved [27].

In men undergoing treatment for a first TB episode, the mortality rate in the first month after starting treatment was highest amongst HIV positive patients receiving ART. We observed a sharp decline in mortality after the first month in this group, although the mortality rate over six months was comparable to those not receiving ART. This may be due to patients at more advanced stages of HIV infection and with lower immunity being selected for ART, or a consequence of IRIS [28]. Other studies have clearly shown the benefits of ART on long term mortality in TB patients, with large reductions in overall mortality in those receiving ART [29],[30]. While this population-based cohort study had excellent follow-up information for the primary outcome of death, it did not have detailed, individual-level clinical data, such as CD4 counts, viral load, treatment regimens, adherence, or interruptions, which would be needed to fully interpret the impact of ART. This might be better ascertained using prospective clinical cohorts, which collect such data routinely.

Mortality rates declined with time after starting TB treatment in all groups, with this effect most appreciable in the HIV negative/unknown group. This is likely to reflect effective treatment in patients with true TB disease. Others have reported that most deaths in HIV positive men undergoing TB treatment were from opportunistic infections (mainly cryptococcus) and occurred later in treatment [18],[19]. Likewise, even amongst men with HIV and confirmed or probable TB, we found at cardiorespiratory autopsy that TB was the cause of 58% of deaths in the first month of treatment but only 27% of deaths in later months. In this study, PCP and bacterial pneumonia together accounted for over one third (46/121) of deaths on the TB programme among men with confirmed or probable TB. We know that other respiratory conditions are often overlooked in TB patients [31]-[33], and that co-trimoxazole prophylaxis substantially reduces mortality from AIDS-related opportunistic infections in SSA settings [34]-[36], and is recommended by South African TB guidelines [25]. Although the HIV programme recommended co-trimoxazole (960 mg daily) for all individuals with a CD4 count of <250 cell/μl and isoniazid (300 mg daily for 6 months) for all newly registered HIV patients, prescribing data were not available for this study. Together, these data also highlight the importance of autopsies, not least to review clinical performance within a TB programme [6],[22].

Two other studies make comparisons between timing of death for first episode and retreated TB [9],[37]. In Malawi, 41% of deaths during first TB episodes occurred in the first month of treatment, compared to 29% for retreatment, but monthly mortality rates were not presented [9]. In India, retreatment was associated with higher mortality (relative risk = 1.93), but survival analysis indicated divergence in mortality between first and retreatment episodes only after three months of treatment [37]. In our study, although there was no difference in the proportion of deaths occurring during the first month of treatment for first and retreatment episodes (23% in both), we observed a significant difference in early mortality rates between the two groups (2.3/100 pm versus 4.7/100 pm, adjusted IRR = 1.76), and our findings are consistent with the overall higher mortality in retreated cases found in other TB programmes [17],[37]. Recurrent TB is common among HIV positive patients in SSA, and can be due to treatment failure, drug resistance, or reinfection. Recurrence may be diagnosed earlier by improved follow-up for patients completing TB treatment, and reduced by isoniazid prophylaxis and ART [38].

In all patient groups, misdiagnosis is a serious concern. Throughout SSA many deaths occur in patients mistakenly treated for TB [13],[20],[21],[33],[39],[40]. In this TB programme, among those with confirmed or probable TB, only 50% (3/6) of deaths at autopsy in men not known to have HIV and 34% (39/115) in HIV positive men could be attributed to TB, whereas clinicians attributed the cause of death to TB in 70% (7/10) and 65% (141/217) of deaths respectively. Even in the first month of treatment among men with confirmed or probable TB, only 58% (15/26) of deaths were attributable to TB at autopsy. Linked to this, and supporting previous findings [39], we found that mortality was strongly associated with diagnostic certainty, although we acknowledge that diagnosis might be complicated by HIV infection and the increased mortality found in “possible” TB cases might be attributable to smear-negative or extrapulmonary TB as well to other undiagnosed infections. It is likely that many men in this programme were inappropriately diagnosed and treated for TB from the outset, indicating preventable deaths and the importance of performing proper diagnostic tests for TB [40].

This study is strengthened by statistical comparisons of incident mortality, presented in conjunction with case fatality, which assists inter-study comparisons because most studies present one but not both statistics. The study benefits from detailed company records showing treatment outcomes for a large cohort but, for this reason, includes only men employed at a platinum mine, which may limit generalisability. Unlike goldminers, who have an increased risk of TB due to silica dust exposure, silicosis is not common in platinum miners, so the findings are relevant to other hospital or occupational cohorts [41],[42]. The study is also strengthened by the availability of cardio-respiratory autopsy data in many patients. The autopsy data are likely to be representative of all deaths in the programme, since the decision to conduct an autopsy was independent of the clinical cause of death. Although rich in mortality and autopsy data, the study is limited by a lack of clinical data, which might provide further insight into some findings. For example, information about TB drug resistance and HIV disease progression was not available, and some data were missing, including dates of HIV testing and HIV negative tests, smear and culture data prior to 2000, and the site of TB infection. We recoded the HIV status of some men based on their cause of death, which may introduce a bias, but we did so to avoid overestimating the mortality in men otherwise thought to be HIV negative. We note that the South African guidelines on regimens for retreatment of TB and ART eligibility changed after the end of the study period. Finally, this study does not include undiagnosed TB cases and there may be many TB deaths occurring outside of the programme, which we have previously shown to be an issue [21].

## Conclusions

We found high mortality in the first month after starting TB treatment in all patient groups, and we have used autopsy data to document the high frequency of non-TB deaths in a large TB programme. TB prevention and earlier accurate diagnosis, recognition of the role of HIV and shorter pathways to TB treatment initiation may reduce death rates. These findings suggest that resources should continue to be targeted to improve diagnostic accuracy and ensure effective clinical care in the early stages of TB treatment.

## Funding

The work was supported by the Colt Foundation (CF/04/08). NF is supported by an NIHR Academic Clinical Lectureship and ML is supported by an Australian Government NHMRC Sidney Sax Early Career Fellowship.

## Authors’ original submitted files for images

Below are the links to the authors’ original submitted files for images. Authors’ original file for figure 1 Authors’ original file for figure 2
