# Supplementary figures and images for: Timing, rates, and causes of death in a large South African tuberculosis programme
Source: BMC Infect Dis. 2014 Dec 21;14:3858. doi: 10.1186/s12879-014-0679-9 (PMC4297465; doi:10.1186/s12879-014-0679-9)

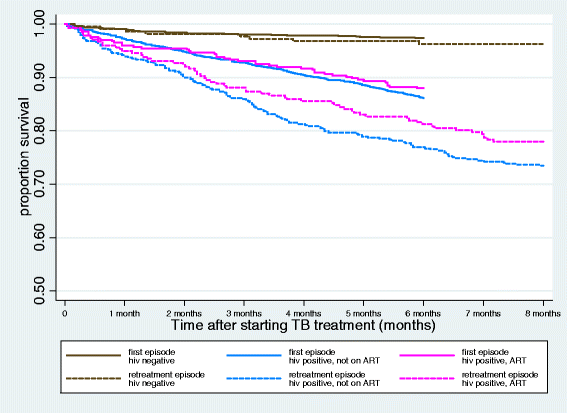

Supplement: Supplementary file 1 — Authors’ original file for figure 1 [file 12879_2014_679_MOESM1_ESM.gif]

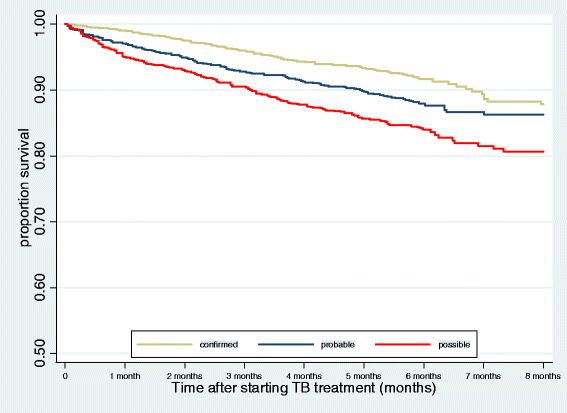

Supplement: Supplementary file 2 — Authors’ original file for figure 2 [file 12879_2014_679_MOESM2_ESM.gif]
